# Model-based PEEP optimisation in mechanical ventilation

**DOI:** 10.1186/1475-925X-10-111

**Published:** 2011-12-23

**Authors:** Yeong Shiong Chiew, J Geoffrey Chase, Geoffrey M Shaw, Ashwath Sundaresan, Thomas Desaive

**Affiliations:** 1 Department of Mechanical Engineering, University of Canterbury, New Zealand; 2 Department of Intensive Care, Christchurch Hospital, New Zealand; 3 Thermodynamics of Irreversible Processes, Institute of Physics, University of Liège, Belgium

**Keywords:** ARDS, ALI, Elastance, Compliance, PEEP, Critical care, Mechanical Ventilation

## Abstract

**Background:**

Acute Respiratory Distress Syndrome (ARDS) patients require mechanical ventilation (MV) for breathing support. Patient-specific PEEP is encouraged for treating different patients but there is no well established method in optimal PEEP selection.

**Methods:**

A study of 10 patients diagnosed with ALI/ARDS whom underwent recruitment manoeuvre is carried out. Airway pressure and flow data are used to identify patient-specific constant lung elastance (*E_lung_*) and time-variant dynamic lung elastance (*E_drs_*) at each PEEP level (increments of 5*cmH_2_O*), for a single compartment linear lung model using integral-based methods. Optimal PEEP is estimated using *E_lung _*versus PEEP, *E_drs_*-Pressure curve and *E_drs _*Area at minimum elastance (maximum compliance) and the inflection of the curves (diminishing return). Results are compared to clinically selected PEEP values. The trials and use of the data were approved by the New Zealand South Island Regional Ethics Committee.

**Results:**

Median absolute percentage fitting error to the data when estimating time-variant *E_drs _*is 0.9% (IQR = 0.5-2.4) and 5.6% [IQR: 1.8-11.3] when estimating constant *E_lung_*. Both *E_lung _*and *E_drs _*decrease with PEEP to a minimum, before rising, and indicating potential over-inflation. Median *E_drs _*over all patients across all PEEP values was 32.2 *cmH_2_O/l *[IQR: 26.1-46.6], reflecting the heterogeneity of ALI/ARDS patients, and their response to PEEP, that complicates standard approaches to PEEP selection. All *E_drs_*-Pressure curves have a clear inflection point before minimum *E_drs_*, making PEEP selection straightforward. Model-based selected PEEP using the proposed metrics were higher than clinically selected values in 7/10 cases.

**Conclusion:**

Continuous monitoring of the patient-specific *E_lung _*and *E_drs _*and minimally invasive PEEP titration provide a unique, patient-specific and physiologically relevant metric to optimize PEEP selection with minimal disruption of MV therapy.

## 1 Introductions

Acute respiratory distress syndrome (ARDS) and acute lung injury (ALI), occurs due to severe inflammatory response of the lung, resulting in direct alveolar injury, pulmonary oedema and alveolar collapse [1,2]. The lung injury greatly impairs the patients breathing, reducing alveolar gas exchange, resulting in possible mortality and morbidity if not given a proper treatment. ALI/ARDS patients are associated with high morbidity, mortality up to 60% [3] and significant medical cost [4].

Patients diagnosed with ALI/ARDS are mechanically ventilated for breathing support [5,6]. Various mechanical ventilation (MV) modes have been introduced to clinicians for the support of patients with ALI/ARDS [7]. However, the fundamentals of MV remains in selecting an optimal positive end-expiratory pressure (PEEP) to maximise patients' lung recruitment, prevent alveoli collapse, and avoid ventilator induced lung injury (VILI) [8]. The heterogeneity of the disease and patients' variable response to MV, encourages PEEP treatment to be patient-specific and individualised. However, there is no gold standard method in PEEP selection; consequently, optimising patient-specific PEEP in MV remains a challenge for clinicians [9-11].

Model-based and patient-specific approaches offer the ability to identify intra- and inter-patients variability and thus, potential to guide MV therapy based on patient's condition and needs [12,13]. This approach provides the opportunity to balance risk of lung injury and lung function support and reduce work of breathing [14] during MV. However, to date, only a few have been tested [15-17] and their potential in critical care is not yet validated.

This research presents several model-based approaches to identify patient-specific disease state and patient-specific response to MV therapy using patient-specific, constant lung elastance (*E_lung_*) [16,18] with comparison of dynamic lung elastance (*E_drs_*) in ALI/ARDS. Dynamic lung elastance (*E_drs_*) is a time-variant lung elastance during each breath in MV. *E_lung _*and *E_drs _*are thus proposed for guiding PEEP selection. By monitoring both the identified parameters (Elastance = 1/Compliance) through limited PEEP titration, it is possible to identify PEEP settings that maximize recruitment, minimize work of breathing without inducing lung injury.

## 2 Methods

### 2.1 Study Design

Ten patients in the Intensive Care Unit (ICU), Christchurch Hospital, New Zealand, diagnosed with ALI or ARDS (*PaO_2_/FiO_2 _*(PF ratio) between 150-300 mmHg), underwent a modified protocol-based recruitment manoeuvre (RM) [17]. PEEP is increased with increments of 5*cmH_2_O *from zero PEEP (ZEEP) until peak airway pressure reaches a limit of 45*cmH_2_O *[19]. Patients were sedated and paralyzed with muscle relaxants to prevent spontaneous breathing efforts. All patients were ventilated using Puritan Bennett PB840 ventilators (Covidien, Boulder, CO, USA) with volume control (tidal volume, *V_t _*= 400~600*ml*), synchronized intermittent mandatory ventilation (SIMV) mode, throughout the trial. The clinical trials and the use of the data were approved by the New Zealand, South Island Regional Ethics Committee.

A heated-pneumotachometer with Hamilton Medical flow sensor (Hamilton Medical, Switzerland) connected to the ventilator circuit Y-piece is used to record patient's airway pressure and flow data. A Dell™ (Dell, Austin, TX, USA) laptop was used in conjunction with National Instruments USB6009 and Labview Signal Express (National Instruments, Austin, TX, USA) to obtain measurements at a sampling rate of 100 Hz. Analysis was performed using MATLAB (The Mathworks, Natick, Massachusetts, USA).

### 2.2 Model-based Analysis

The model-based approach incorporates a physiologically relevant and validated recruitment model [17,20] with the use of a single compartment linear lung model that captures fundamental lung mechanics and properties in real-time to identify patient-specific constant lung elastance (*E_lung_*) and dynamic lung elastance (*E_drs_*) during MV. The model uses transpulmonary pressure (*P_tp_*), volume (*V*) and flow (*Q*) and offset pressure (*P_0_*), to identify lung elastance (*E_lung_*) and resistance (*R_lung_*). Patient-specific lung elastance, *E_lung _*reflects the lung stiffness (1/Compliance). Therefore, a lower *E_lung _*is a more compliant lung. *E_lung _*is identified from measured data using an integral-based method [21]. The model is defined:

(1) $$P_{tp}=E_{lung}V+R_{lung}Q+P_{0}$$

Airway pressure is related to transpulmonary pressure (*P_tp_*) and pleural pressure (*P_pl_*) by:

(2) $$P_{tp}=P_{aw}-P_{pl}$$

When the patient is sedated and fully dependant on the ventilator to breathe, it can be assumed that there is no chest wall activity, allowing *P_pl _*to be omitted in this case. Equation 1 is then further modified to eliminate *P_pl_*, yielding:

(3) $$P_{aw}=E_{lung}V+R_{lung}Q+P_{0}$$

Patient-specific dynamic lung elastance, *E_drs_*, is identified as a time-variant lung elastance and Equation (3) is defined:

(4) $$P_{aw}\left(t\right)=E_{drs}\left(t\right)V\left(t\right)+R_{lung}Q\left(t\right)+P_{0}$$

To ensure that the identified parameters of constant *E_lung _*and time-variant *E_drs _*(*E_drs_(t)*) are valid, the absolute percentage error between the identified model and measured clinical pressure data is reported.

### 2.3 Model-Based PEEP Selection

During each breathing cycle, as PEEP rises, lung elastance (*E_lung_*) falls as new lung volume is recruited faster than the pressure build-ups in the lung. If little or no recruitment occurs, *E_lung _*rises with PEEP indicating that pressure above that PEEP level was unable to recruit significant new lung volume and is, instead, beginning to stretch already recruited lung [22]. Hence, recruitment and potential lung injury can be balanced by selecting PEEP at minimum *E_lung_*.

Compared to a single, constant *E_lung _*value at each PEEP, identifying time-variant *E_drs _*allows this change to be seen dynamically within each breath as pressure increases thus allowing a more detail view of patient's lung physiological condition. Three model-based approaches based on patient-specific *E_lung _*and *E_drs _*trajectory in a patient's breath at different PEEP levels are used to optimize PEEP selection.

**Minimum *E_drs _*and *E_lung_***: locates the point where minimum *E_drs _or E_lung _*occurs over all PEEP values (and pressure for *E_drs_*) during the recruitment manoeuvre.

**Minimum *E_drs _*Area**: *E_drs _*Area is obtained by integrating *E_drs _*over time during the patient's breathing cycle at each PEEP. *E_drs _*Area is more clinically relevant than median or mean *E_drs _*throughout each breath and can be shown to be proportional to patient-specific work of breathing.

**Inflection Method**: This method detects the inflection in the *E_drs _*Area-PEEP and *E_lung_*-PEEP curves. Inflection is defined here at the PEEP value with *E_drs _*value 5-10% above (before) minimum *E_drs _*Area or *E_lung _*(105~110% of minimum *E_drs _*Area or *E_lung_*). PEEP is selected where inflection occurs, as a point of diminishing returns.

The overall approach implies that as long as *E_drs _*falls during each breath, as PEEP level increases, that recruitment of new volume outweighs lung stretching as flow and volume follow a path of lesser or least resistance. These methods are thus attempts to maximize recruitment (Minimum *E_drs _*and Minimum *E_drs _*Area) and also ensure safety from excessive pressure (Inflection Method). These metrics are three of many possibilities to demonstrate the concept.

### 2.4 Edrs Area and Work of Breathing

These approaches were also compared with selecting PEEP using the identified minimum or inflection of constant *E_lung_*, for comparison to other similar work [23]. Patient-specific *E_lung _*and *E_drs _*are only analyzed during inspiration and not during the expiratory cycle. This choice was made because increases in pressure induce lung damage as it passes a limit and thus expiration (decreasing pressure) should not be used to guide PEEP selection.

A higher resolution of the trend changes in *E_drs _*can be observed using *E_drs _*Area. *E_drs _*Area is obtained through integration of *E_drs _*with time. It is also known that the work of breathing (*WOB*) [24,25] for a patient is proportional to lung elastance. In general, more work is required to fill a given lung volume with higher elastance. *WOB *is defined:

(5) $$WOB=P_{aw}\times V$$

Substituting *P_aw _*from Equation (3) into Equation (5) and using *P_0 _*= 0, (atmospheric).

(6) $$\begin{array}{lll} WOB & =\left(E_{lung}V+R_{lung}Q\right)\times V\; & \\ & =E_{lung}V^{2}+R_{lung}QV\; & \\ & \; & \end{array}$$

From Equation (6), work of breathing can be divided into work to overcome lung elastance (*WOB_E _*= *E_lung_V^2^*) and work to overcome airway resistance (*WOB_R _*= *R_lung_QV*). Substitution of dynamic lung elastance, *E_drs_*, for constant *E_lung _*enables a derivation for *WOB_E_*:

(7) $$E_{drs}=WOB_{E}\left(t\right)∕V{\left(t\right)}^{2}$$

*E_drs _*Area in Equation (8) is the integral of Equation (7), yielding the relation of *E_drs _*to the work of breathing required to overcome lung elastance at a given level of PEEP and mode of MV.

(8) $$E_{drs}Area=\int E_{drs}\left(t\right)dt$$

### 2.5 Analysis and Comparisons

In this study, *E_lung _*and median *E_drs _*are compared using Pearson's linear correlation coefficients to relate these metrics. *E_lung _*and *E_drs _*Area are also compared to median *E_drs _*and *WOB_E _*to ensure there was no loss of information for each patient at different PEEP values, and to show the validity of Equation (7) and using *E_drs _*Area. Finally, clinically selected PEEP is compared to the value determined by proposed model-based metrics.

## 3 Results

Table 1 shows the clinical details of the 10 patients recruited with their clinical diagnostics, and PF ratios. Table 2 shows the median [Inter-quartile Range (IQR)] *E_drs _*for each patient and PEEP, and absolute percentage fitting error. Median absolute percentage fitting error (APE_Edrs(t)_) across all patients and PEEP is 0.9*% *[IQR: 0.5-2.4]. Median *E_drs _*at each PEEP is 32.2*cmH_2_O/l *[IQR: 26.1-46.6]. Median [IQR] *E_drs _*decreases with increasing PEEP until the minimum *E_drs_*. Patients who suffer from COPD (Patients 1, 4, 5, 9 and 10) have significantly higher *E_drs _*than others (P < 0.0001), as expected clinically. Table 3 shows the constant lung elastance (*E_lung_*) at each PEEP with median = 32.2*cmH_2_O/l *[IQR: 25.0-45.9], and absolute percentage fitting (APE_Elung_) at 5.6% [IQR: 1.8-11.3]. Table 4 shows the *E_drs _*Area at each PEEP with median [IQR] of 34.0*cmH_2_Os/l *[IQR: 24.7-48.5].

**Table 1:** Patient demography.

*Patients*	*Sex*	*Age (year)*	*Clinical Diagnostic*	*PF Ratio*
**1**	F	61	Peritonitis, COPD	214
**2**	M	22	Trauma	180
**3**	M	55	Aspiration	222
**4**	M	88	Pneumonia, COPD	165
**5**	M	59	Pneumonia, COPD	285
**6**	M	69	Trauma	280
**7**	M	56	Legionnaires	265
**8**	F	54	Aspiration	302
**9**	M	37	H1N1, COPD*	182
**10**	M	56	Legionnaires, COPD	237

*Chronic Obstructive Pulmonary Disease

**Table 2:** Patient-specific dynamic lung elastance (*E_drs_*) at each PEEP level.

Patient	Dynamic Lung Elastance, *E_drs _*(*cmH_2_O/l*) Median [IQR]							*E_drs _(cmH_2_O/l)* *Median [IQR]*	APE* *(%)* *Median [IQR]*
		
	PEEP (*cmH_2_O*)								
		
	0	5	10	15	20	25	30		
**1**	63.1 [46.9-114.9]	53.8 [43.0-80.2]	43.6 [38.4-54.5]	35.0 [33.3-39.4]	33.4 [32.0-34.2]	31.1 [32.0-32.4]	PEEP 27 32.2 [31.9-32.6]	35.0 [32.5-51.2]	1.1 [0.5-4.1]
**2**	30.8 [26.3-45.1]	26.4 [23.7-31.4]	23.1 [22.0-24.3]	22.1 [22.0-22.6]	22.5 [22.4-22.6]	PEEP 22 23.1 [22.9-23.2]		23.1 [22.5-26.4]	0.7 [0.6-2.4]
**3**	26.9 [22.6-36.9]	22.1 [20.2-25.6]	18.3 [18.0-19.0]	17.3 [17.2-17.4]	17.5 [17.1-17.5]	17.8 [17.4-18.7]	PEEP 28 19.2 [17.9-19.7]	18.3 [17.6-21.4]	0.6 [0.5-1.3]
**4**	73.2 [50.4-144.4]	70.4 [49.9-126.9]	54.5 [41.7-82.3]	36.8 [30.6-43.9]	28.5 [25.6-31.4]	25.9 [21.6-28.4]	23.1 [19.4-25.5]	36.8 [26.6-66.4]	3.4 [0.9-5.4]
**5**	105.7 [80.6-199.8]	97.8 [77.5-166.8]	89.3 [74.3-143.4]	79.4 [68.6-107.3]	67.3 [61.4-79.4]	52.3 [52.0-55.8]		84.4 [67.3-97.8]	3.2 [0.9-6.0]
**6**	30.4 [25.9-39.1]	26.2 [25.5-27.2]	23.3 [22.4-23.5]	21.6 [21.5-21.8]	21.8 [21.3-22.5]	23.3 [22.6-23.9]		23.3 [21.8-26.2]	0.8 [0.6-1.2]
**7**	49.3 [46.1-62.4]	42.2 [41.5-43.1]	44.3 [41.8-47.7]	53.6 [48.8-59.7]	PEEP 16 52.4 [50.3-57.6]			49.3 [43.8-52.7]	1.6 [1.3-2.0]
**8**	45.7 [37.9-67.8]	37.2 [32.9-43.0]	31.8 [29.9-33.5]	28.8 [28.0-29.8]	27.4 [27.1-27.9]	26.8 [26.3-27.0]	27.0 [26.8-27.5]	28.8 [27.1-35.9]	0.8 [0.5-2.2]
**9**	58.1 [47.1-100.8]	40.5 [36.4-52.8]	39.9 [35.8-48.7]	31.2 [30.2-33.6]	28.3 [27.9-29.0]	26.3 [26.3-26.5]	26.2 [25.8-26.5]	31.2 [26.8-40.4]	0.8 [0.4-2.1]
**10**	54.4 [48.1-76.2]	45.2 [41.9-51.8]	39.4 [38.4-41.7]	35.9 [35.7-36.0]	33.9 [33.7-34.1]	33.9 [33.4-34.6]	PEEP 27 33.9 [33.2-34.8]	35.9 [33.9-43.8]	0.4 [0.4-0.9]

**Median** **[IQR]**	51.9 [30.8-63.1]	41.4 [26.4-53.8]	39.7 [23.3-44.3]	33.1 [22.1-36.8]	28.4* [22.5-33.9]	26.3* [23.1-32.2]	26.6* [23.1-32.2]	32.2 [26.1-46.6]	0.9 [0.5-2.4]

*APE - Absolute Percentage Fitting Error (%)

*Values presented include value from different PEEP.

**Table 3:** Patient-specific constant lung elastance (*E_lung_*) at different PEEP.

Patient	Constant Lung Elastance, *E_lung _*(*cmH_2_O/l*)							*E_lung _(cmH_2_O/l)* Median [IQR]	*APE* *(%)* Median [IQR]
		
	PEEP (*cmH_2_O*)								
		
	0	5	10	15	20	25	30		
**1**	53.8	47.0	41.2	32.8	32.8	32.1	PEEP 27 32.2	34.7 [32.4-45.5]	7.2 [1.7-19.0]
**2**	27.7	25.3	22.8	22.3	22.6	PEEP 22 23.1		23.0 [22.6-25.3]	2.5 [1.1-7.7]
**3**	24.0	21.6	18.3	17.3	17.4	18.1	PEEP 28 19.1	18.3 [17.6-20.9]	4.2 [1.6-6.6]
**4**	60.2	59.7	50.1	35.1	27.8	25.3	22.5	35.1 [25.9-57.3]	17.7 [15.4-32.1]
**5**	87.4	84.0	81.2	74.3	65.7	53.1		77.8 [65.7-84.0]	15.7 [9.2-19.8]
**6**	27.1	25.5	22.8	21.6	21.8	23.4		23.1 [21.8-25.5]	2.7 [2.2-4.2]
**7**	47.7	42.5	45.5	55.7	PEEP 16 55.3			47.7 [44.8-55.4]	6.2 [5.0-7.7]
**8**	41.7	35.5	31.2	28.7	27.5	26.6	27.0	28.7 [27.2-34.4]	2.9 [1.3-8.7]
**9**	51.3	39.1	38.2	31.1	28.2	26.2	26.1	29.7 [26.2-38.7]	3.1 [1.0-10.8]
**10**	51.0	44.1	39.2	35.8	33.9	34.0	PEEP 27 34.2	35.8 [34.1-42.9]	2.0 [1.0-5.6]

**Median [IQR]**	49.4 [27.7-53.8]	40.8 [25.5-47.0]	38.7 [22.8-45.5]	31.9 [22.3-35.5]	28.0* [22.6-33.9]	26.2* [23.3-32.6]	26.6* [22.5-32.2]	32.2 [25.0-45.9]	5.6 [1.8-11.3]

Eg. PEEP 16 is included in PEEP 20 Median [IQR]

*Values presented include value from different PEEP. Eg. PEEP 16 is included in PEEP 20 Median [IQR]

**Table 4:** Patient-specific *E_drs _*Area at different PEEP.

Patient	*E_drs _Area *(*mH_2_Os/l*)							*E_drs _Area *(*cmH_2_Os/l*) Median [IQR]
		
	PEEP (*cmH_2_O*)							
		
	0	5	10	15	20	25	30	
**1**	84.6	49.5	37.1	28.9	26.6	25.7	PEEP 27 25.7	28.9 [25.9-46.4]
**2**	34.0	24.8	21.0	20.2	20.3	PEEP 22 20.7		20.9 [20.3-24.8]
**3**	37.7	27.6	22.2	20.8	19.1	19.7	PEEP 28 18.9	20.8 [19.3-26.3]
**4**	102.2	91.2	61.7	37.9	31.7	48.1	47.5	48.1 [40.3-83.8]
**5**	118.7	99.9	89.1	70.6	75.7	42.9		82.4 [70.6-99.9]
**6**	29.4	23.8	20.8	21.6	19.5	20.8		21.2 [20.8-23.8]
**7**	37.6	33.8	31.3	37.9	PEEP 16 32.1			33.8 [31.9-37.7]
**8**	55.1	38.5	32.0	29.0	27.5	24.1	24.3	29.0 [25.1-36.9]
**9**	106.5	55.2	51.3	38.3	34.1	31.6	31.3	38.4 [32.2-54.2]
**10**	74.7	52.6	44.0	39.5	37.3	37.2	PEEP 27 37.3	39.5 [37.3-50.5]

**Median [IQR]**	64.9 [37.6-102.2]	44.0 [27.6-55.2]	34.6 [22.2-51.3]	33.5 [21.6-38.4]	29.6* [20.3-34.1]	25.7* [20.8-38.6]	28.5* [24.3-37.3]	34.0 [24.7-48.5]

*Values presented include value from different PEEP. Eg. PEEP 16 is included in PEEP 20 Median [IQR]

**Figure 1:**
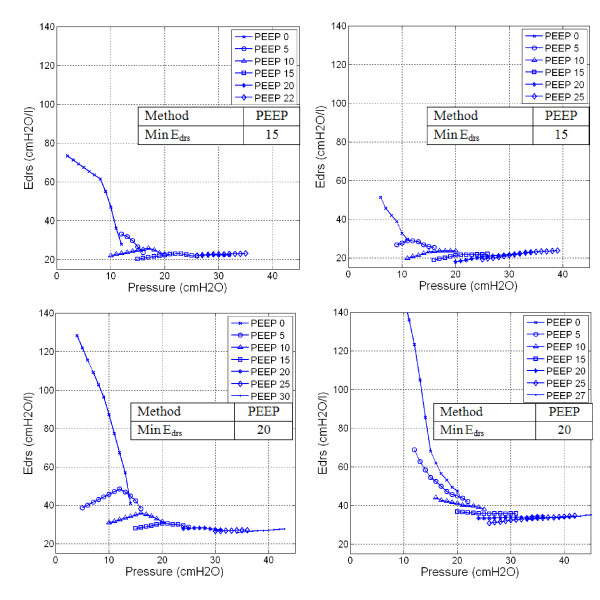
**Dynamic lung elastance (*E_drs_*)-Pressure-PEEP plot**. Top Left Panel: Patient 2, Top Right Panel: Patient 6. Both patients show significant *E_drs _*drop from lower zero PEEP to PEEP 15*cmH_2_O*. Further increase of PEEP to 20*cmH_2_O *shows increase of overall *E_drs_*. Bottom Left Panel: Patient 8, Bottom Right Panel: Patient 10. Both patients show a consistent drop in overall *E_drs _*with increasing of PEEP and overall *E_drs _*did not rise with PEEP for the entire ranged considered.

Figure 1 shows patient-specific time-varying *E_drs _*at each PEEP level for Patients 2, 6, 8 and 10. *E_drs _*decreases as pressure increases at each PEEP. However, at higher PEEP, this trend can reverse indicating stretching exceeding recruitment of new lung volume. The optimal PEEP derived by minimum *E_drs _*is indicated.

**Figure 2:**
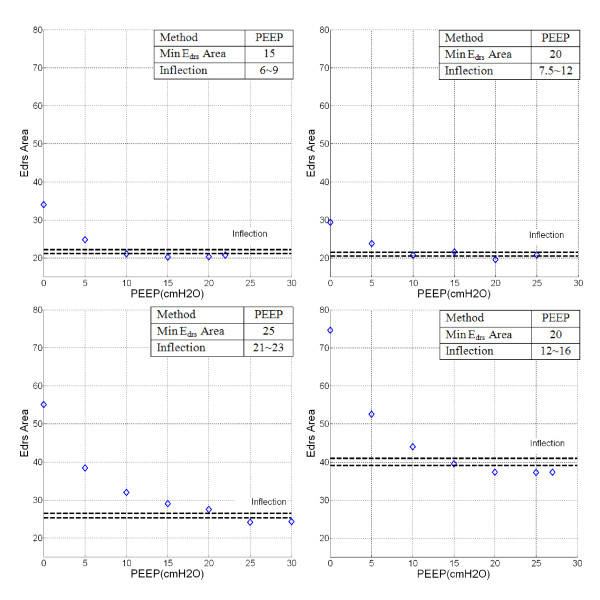
***E_drs _*Area-PEEP plot**. Top Left Panel: Patient 2, Top Right Panel: Patient 6. Bottom Left Panel: Patient 8, Bottom Right Panel: Patient 10. Severe COPD or patients with similar clinical features (e.g. Patient 10) showed significantly higher *E_drs _*Area compared to other patients. PEEP selection is based on minimum *E_drs_*-Area and the inflection method with PEEP increase.

Figure 2 shows patient-specific *E_drs _*Area for Patients 2, 6, 8 and 10 with PEEP. The optimal PEEP is derived using minimum *E_drs _*Area and Inflection method with the band of 5-10% above minimum *E_drs _*Area shown by the dashed-lines.

**Figure 3:**
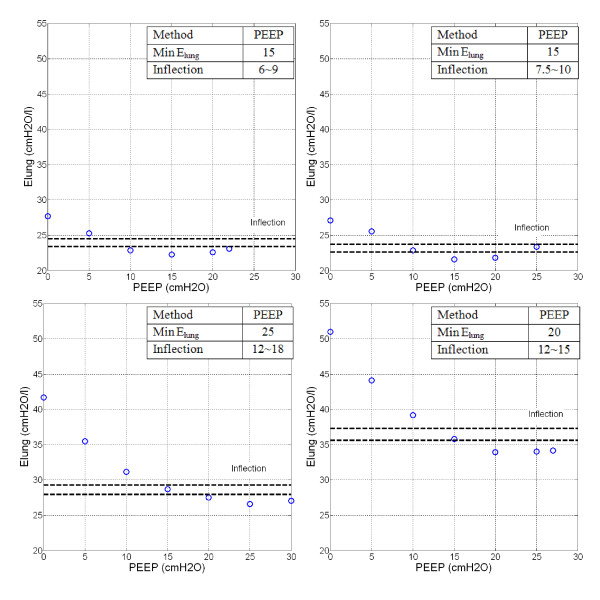
***E_lung_*-PEEP plot**. Top Left Panel: Patient 2, Top Right Panel: Patient 6. Bottom Left Panel: Patient 8, Bottom Right Panel: Patient 10. PEEP derived from Minimum *E_lung _*and Inflection method are as indicated.

Figure 3 shows patient-specific constant lung elastance (*E_lung_*) with increasing PEEP for Patients 2, 6, 8 and 10. *E_lung _*decreases with PEEP and the trend is similar to the *E_drs _*Area-PEEP plot of Figure 2, as expected from the high correlation. The optimal PEEP using minimum *E_lung _*and Inflection *E_lung _*(Dashed-lines) are also indicated.

**Figure 4:**
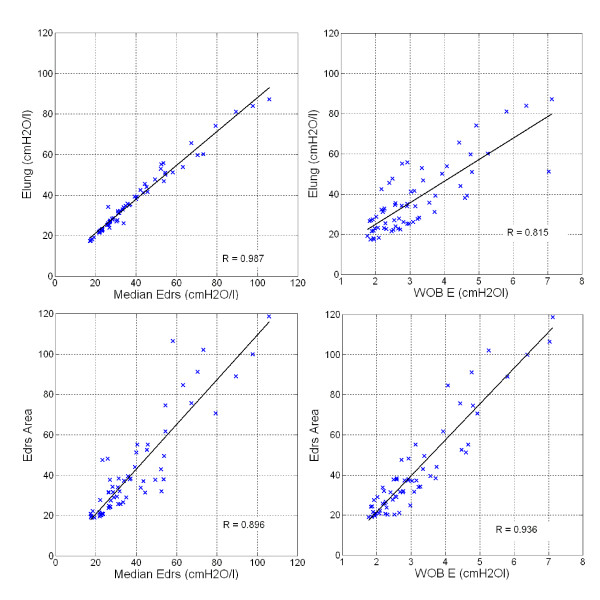
**Pearson's Correlation**. Top Left Panel: *E_lung_*-Median *E_drs_*, R = 0.987. Top Right Panel: *E_lung_*-*WOB_E_*, R = 0.815. Bottom Left Panel: *E_drs _*Area-Median *E_drs_*, R = 0.896. Bottom Right Panel: *E_drs _*Area-*WOB_E_*, R = 0.936.

Across all 10 patients, patient-specific constant lung elastance (*E_lung_*) can be represented by the median of dynamic lung elastance (*E_drs_*) with correlation R = 0.987. Correlation of *E_lung _*and *WOB_E _*is R = 0.815. *E_drs _*Area and median *E_drs _*are also closely correlated with R = 0.896. Hence, *E_drs _*can be represented with *E_drs _*Area, where *E_drs _*Area captures all *E_drs _*values in a given breath and thus, is a more physiologically representative metric. Finally, validating Equation (2), *E_drs _*Area is correlated to the work to overcome lung elastance, *WOB_E_*, as expected, with R = 0.936. The correlations are shown in Figure 4.

Table 5 compares clinically selected PEEP during MV therapy with PEEP selected using Minimum *E_drs_*, and Minimum *E_drs _*Area and the Inflection method. The clinical values are set over a much narrower range, both higher and lower than those selected using *E_drs_*. Minimum *E_drs _*Area always selects a higher PEEP, by definition, than the Inflection method. However, Minimum *E_drs _*Area selects PEEP similar to or higher than Minimum *E_drs_*, where it also thus adds consideration of the reduction in overall *WOB_E _*in selecting PEEP. PEEP derived from minimum *E_lung _*and Inflection *E_lung _*are also indicated.

**Table 5:** PEEP (*cmH_2_O*) selection in clinical and model-based approach.

	Patients									
	
Selection Methods	1	2	3	4	5	6	7	8	9	10
**Clinical**	10	12	10	10	12	11	7.5	12	10	10

**Minimum *E_drs _***	20	15	15	25	25	15	5	20	15	20

**Minimum *E_drs _*Area**	25	15	20	20	25	20	10	25	25	20

**Inflection** ***E_drs _*Area**	14~16	6~9	15~17	16~18	22~24	7.5~12	5~7.5	21~23	20~23	12~16

**Minimum *E_lung_***	25	15	15	30	25	15	5	25	30	20

**Inflection** ***E_lung_***	13~17	6~9	8~10	26~27	21~24	7.5~10	5	12~18	19~22	12 ~15

## 4 Discussion

### 4.1 Model-based PEEP Selection

Median fitting error for time-variant *E_drs _*in Table 2 is less than 1%, showing that a single compartment lung model can be used for time-varying *E_drs _*estimation. The wide range of patient-specific *E_drs _*across all patients and PEEP shown in Table 2 reflects the heterogeneity of ALI/ARDS patient condition and response to PEEP that makes standardising and PEEP selection difficult [26]. Compared to the estimation of *E_lung _*in Table 3, median fitting error is 5.6% and in specific cases, fitting error can be as high as 15.7-17.7% (Patients 4 and 5). This latter result indicates that a first order model can be used to estimate most patient-specific constant *E_lung_*, but, in several cases, the model may not accurately represent patients' physiological condition. Time-varying *E_drs _*provides a better model fit across all patients and also provides a clearer insight into the patient's physiological condition, and is thus the better model-based metric.

Figures 1 and Figure 2 shows *E_drs_*-Pressure-PEEP curves and *E_drs _*Area decrease with increasing PEEP, lung pressure, and volume over each breath. In the beginning of the recruitment manoeuvre, at zero end-expiratory pressure (ZEEP), *E_drs _*is relatively very high for all patients with median 51.9*cmH_2_O/l *[IQR: 30.8-63.1]. In particular, chronic obstructive pulmonary disease (COPD) patients or patients with similar clinical features [27] (Patients 1, 4, 5, 9 and 10) have initially the highest *E_drs _*median, as expected, from 63.1*cmH_2_O/l *[IQR: 57.2-81.3] versus 30.8*cmH_2_O/l *[IQR: 29.5-46.6] for the other patients (p = 0.0079). As PEEP rises, it is observed that *E_drs _*curves drop at patient-specific rates. High constant lung elastance, *E_lung _*at ZEEP and decreasing elastance as PEEP increments are also observed in Figure 3 for Patient 10.

In all cases, patient-specific *E_drs _*and *E_lung _*decrease to a patient-specific minimum before increasing at higher PEEP. Minimum *E_drs _*and *E_lung _*suggest the point where the lung is most compliant, if ventilated at that PEEP level. Further increases in PEEP and pressure thus lead to increased *E_lung _*or *E_drs_*, and thus increase detrimental effects. In particular, increases in *E_lung _*or *E_drs _*can be associated with overstretching of the patient's lung [16,28]. However, the heterogeneity of ALI/ARDS means there is a possibility of overstretching of healthy lung units even at low PEEP and airway pressures [10]. Thus, Minimum or, perhaps preferably, Inflection *E_drs _*and *E_lung _*can provide a potentially higher resolution metric.

Patients 2 and 6 (Figure 1, 2, 3: Top panels) are examples where patient-specific *E_drs_*, *E_drs _*Area and *E_lung _*increase after descending to a minimum. Results suggest that further increases of PEEP and inflation pressures will stretch lung units causing possible damage, as seen by increasing *E_drs _*at higher PEEP. The rise of *E_drs _*occurs at relatively low PEEP and pressure 15-20*cmH_2_O *in these two patients.

In contrast, Patients 8 and 10 (Figure 1, 2, 3: Bottom panels) never see *E_drs _*or *E_lung _*rising even at the maximum PEEP used in this study. However, the *E_drs _*range at higher PEEP for Patients 8 and 10 (PEEP 15~30*cmH_2_O*) is relatively small with median *E_drs _*= 31.3*cmH_2_O/l*, [IQR = 27.2-33.9]. This outcome indicates that further increases of PEEP from 15 to 30*cmH_2_O *has no added advantage in reducing *E_drs_*, suggesting PEEP selection should be made at using the Inflection method.

Table 2 shows median [IQR] *E_drs _*for every patient and PEEP. The IQR range drops significantly for every patient as PEEP increases. This range also indicates lung status or condition with the influence of pressure. A small IQR range indicates that the lung is ventilated at a PEEP level where maximal lung recruitment occurs over a narrow pressure range as tidal volume, *V_t _*is fixed in the MV mode used. A high IQR range shows the opposite. Hence, the lengths along pressure in Figure 1 also indicate how readily the patient was recruited and that easiest recruitment occurs at minimum *E_drs _*[29].

Table 4 shows the patient-specific *E_drs _*Area at each PEEP. It is found that *E_drs _*Area is closely related to median *E_drs_*, as shown in Figure 4. *E_drs _*Area at lower PEEP with median 64.9 cmH2Os/l [IQR: 37.6-102.2] is observed and as PEEP increases, *E_drs _*Area decreases. Upon reaching minimum *E_drs _*Area, patient-specific *E_drs _*Area increase with PEEP (Patients 2, 4, 6, 7 and 10). This trend is similar to the trend observed in patient-specific dynamic *E_drs _*(Table 2) and constant *E_lung _*(Table 3). Optimal PEEP derived using minimum or inflection method in *E_drs _*Area is similar to minimum patient-specific *E_drs _*but different as *E_drs _*Area considers the whole inspiration and the effect of *WOB_E_*. It is also found that *E_drs _*Area is closely correlated to work in overcoming the lung elastic properties (*WOB_E_*). This means that *E_drs _*Area provides combined information of patients-specific lung physiological conditions as well as work of breathing.

Table 5 shows the model-based approaches to PEEP selection compared to clinically selected PEEP. For 9 of 10 patients, the PEEP value selected using Minimum *E_drs _*and *E_drs _*Area results in a value higher than the clinically selected PEEP. This latter result suggests that these patients could be treated at PEEP levels higher than clinically selected PEEP. When Minimum *E_drs _*or *E_drs _*Area metrics are compared with Minimum *E_lung _*[16], they result in selecting similar PEEP. However, selecting PEEP is a trade off in minimizing lung pressure and potential damage, versus maximizing recruitment. Hence, the Inflection method offers similar recruitment at a lower PEEP and may be a safer choice, although its selected values are still higher than clinically selected in 7 of 10 cases. Overall, these results reflect the heterogeneity of the ALI/ARDS lung and the need for patient-specific approaches to select PEEP.

**Figure 5:**
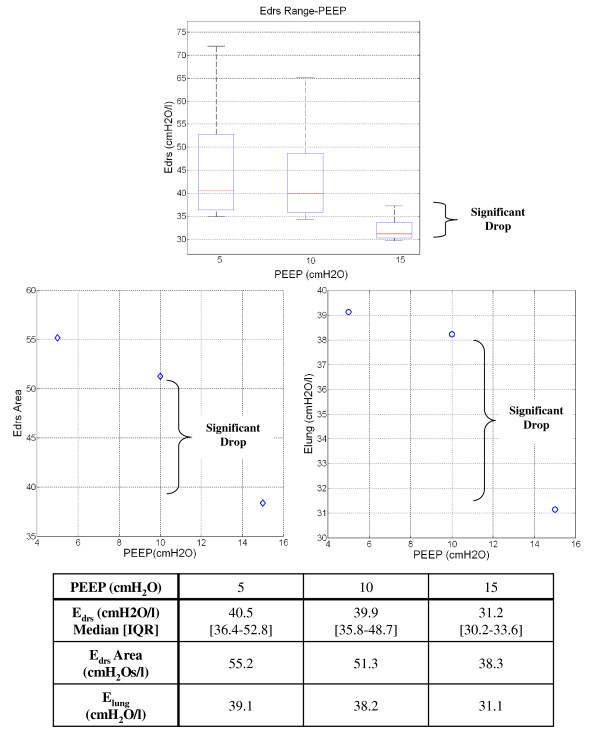
**Patient 9 *E_drs_*, *E_drs _*Area and *E_lung _*change with PEEP**. Top Panel: Box-and-whisker diagram for Patient 9 *E_drs _*when PEEP increase from 5 to 10*cmH_2_O*. The *E_drs _*drops significantly when PEEP is increase from 10 to 15*cmH_2_O*. Bottom Left Panel: *E_drs _*Area-PEEP plot for Patient 9. Bottom Right Panel: *E_lung_*-PEEP for Patient 9.

Patient 9 is an interesting case which illustrates the model's potential to capture unique patient-specific lung recruitment and condition as it occurs in a clinically and physiologically relevant manner. When the patient is ventilated from PEEP of 5 to 10*cmH_2_O*, median *E_drs _*only decreases by less than 1.0*cmH_2_O/l*. However, when PEEP is increased to 15*cmH_2_O*, the median *E_drs _*drops significantly, as shown in Figure 5. This smaller *E_drs _*drop suggests that only minimal lung volume is recruited from PEEP of 5 to 10 *cmH_2_O*. The significant drop in *E_drs _*at PEEP 15*cmH_2_O *indicates that PEEP *15 cmH_2_O *has overcome recruitment resistance and additional new lung volume is recruited. Patient 9 was diagnosed with H1N1 and high PEEP for lung recruitment has proven to be beneficial for these patients [30]. Similar trends can be observed in Figure 5 bottom panels with the *E_drs _*Area-PEEP plot and *E_lung_*-PEEP-plot.

### 4.2 Limitations

In this research, the lung model used to identify patient-specific *E_drs _*comprised a single compartment lung model. It was initially proposed for simple computational analysis and neglects the effect of nonlinear flow [31]. However, this analysis is based predominantly on trend comparisons, where the patient is their own reference. In addition, the model is simple and capable of capturing the fundamental lung mechanics, which varies intra- and inter- patients. Hence, this limitation should be minimal in this case, but should be confirmed with direct prospective clinical studies.

During the clinical trials, the patients were sedated and paralyzed using muscle relaxants. It is assumed that after sedation, the patient will be fully dependant on mechanical ventilation and not have spontaneous breathing effort. This assumption thus assumes the patient's pleural pressure (*P_pl_*) after sedation is zero and allows *P_pl _*in Equation (3) to be omitted, which may not be entirely valid [32]. However, this assumption is made for the first step study to prove the concept within a simpler situation. Otherwise, the terms *E_lung _*and *E_drs _*would represent a respiratory system elastance [31] and time-variant dynamic respiratory system elastance. However, given the low fitting errors observed, this issue should have little impact in this research.

During the course of estimating patient-specific *E_lung _*or *E_drs_*, respiratory system resistance, *R*, is assumed overall constant within a physiological range [33] as PEEP increases. This assumption may not be entirely valid in some cases [33,34]. However, continuous measurements of respiratory resistance are not typically available and the effect of this resistive term is limited mathematically in its impact. Equally, trend comparison, as used here, across PEEP values will reduce the impact.

The identification of *E_lung_*, *E_drs _*and *E_drs _*Area during MV is presented as a method to select PEEP, but there is currently no conclusive, optimum overall *E_drs _*or *E_drs _*Area in patients. *E_drs _*range varies depending on patient disease state and thus will also change over time. However, this trial includes only 10 patients, and there is not yet enough clinical data to indicate an optimum *E_lung_*, *E_drs _*or *E_drs _*Area value for a specific patient or group. On-going, prospective trials with more specific patient groups should develop more conclusive outcomes, relating specific set values of *E_drs _*metrics to effective patient-specific treatments and clinical outcome.

In particular, the time-varying *E_drs _*value and its change over a given breathing cycle, provides additional insight to guide ventilation that is not investigated here. For example, changes in ventilator pattern or mode to modify the *E_drs _*trajectory could also be used with this data to guide therapy choice. However, this study does not have the numbers or design to provide that advice, or specific *E_drs _*values associated with specific decrease state or lung damage.

## 5 Conclusions

The model-based approach presented provides patient-specific, physiological insight not directly measurable without additional invasive, disruptive and clinically intensive test manoeuvres. This method can be directly implemented using modern ventilators with minimal, limited PEEP titrations, and thus without significant interruption to ongoing therapy. In particular, the full manoeuvres used here would not be required for clinical use, and only modest PEEP changes (3-8*cmH_2_O*) would be required to determine if *E_drs _*was decreasing at a different PEEP. *E_drs _*offers higher resolution in patients' response to change of pressure and PEEP, which is potentially, a better metric compared to existing constant lung elastance estimation. Thus, the overall method is readily generalisable and clinical practicable. It is able to capture patient-specific condition and responsiveness to PEEP and recruitment accurately, and as clinically expected. Hence, the approach presented offers significant potential to improve clinical insight and delivery of mechanical ventilation, and should be prospectively tested.

## 6 List of Abbreviations

ALI: Acute lung injury; APE: Absolute percentage error; ARDS: Acute respiratory distress syndrome; COPD: Chronic Obstructive Pulmonary Disease; *E_lung_*: Patient-specific constant lung elastance; *E_drs_*: Patient-specific dynamic lung elastance; *FiO_2_*: Fraction of Inspired Oxygen; ICU: Intensive care unit; IQR: Interquartile Range; MV: Mechanical ventilation; *PaO_2: _*Partial pressure of oxygen in arterial blood; *P_aw_*: Airway pressure; *P_pl: _*Pleural pressure; *P_tp_*: Transpulmonary pressure; PEEP: Positive end expiratory pressure; PF Ratio: *PaO_2_/FiO_2_*; *P_0_*: Offset pressure; *Q*: Flow; RM: Recruitment manoeuvre; *R_lung_*: Resistance; SIMV: Synchronized intermittent mandatory ventilation; *t*: Time; *V*: Volume; VILI: Ventilation induced lung injury; *V_t_*: Tidal volume; *WOB*: Work of Breathing; *WOB_E_*: Work to overcome respiratory system elastance; *WOB_R_*: Work to overcome airway resistance; ZEEP: Zero PEEP

## 7 Competing Interests

The authors declare that they have no competing interests.

## 8 Authors Contribution

YSC, JGC, GMS created and defined the model. YSC, JGC and TD had input to analysis of results. GMS, AS implemented trials clinically with input from all others. All authors had input in writing and revising the manuscript. All authors have read and approved the final manuscript.

## 9 Consent

Written informed consent was obtained from the participant and or relative/friends/family of this study. A copy of written consent is available for review by the Editor-in-Chief of this journal.
